# Variants in the SARS2 gene cause HUPRA syndrome with atypical features: two case reports and review of the literature

**DOI:** 10.1093/omcr/omad119

**Published:** 2023-11-28

**Authors:** Elias Edward Lahham, Juhina Jamal Hasassneh, Dua Osamah Adawi, Mohamad Khaled Ismail

**Affiliations:** Radiation Oncology Department, Augusta Victoria Hospital, Jerusalem, Palestine; Pediatric Department, Beit-Jala Governmental Hospital, Bethlehem, Palestine; Pediatric Department, Beit-Jala Governmental Hospital, Bethlehem, Palestine; Pediatric Department, Beit-Jala Governmental Hospital, Bethlehem, Palestine

**Keywords:** HUPRA syndromeSARS2 geneseryl-tRNA synthetaseprogressive renal failure in infancymitochondrial diseases

## Abstract

Hyperuricemia, pulmonary hypertension, renal failure in infancy, and alkalosis (HUPRA syndrome) is a rare autosomal recessive mitochondrial disease with a prevalence of <1:1 000 000, due to variations in the seryl-tRNA synthetase (SARS2) gene encoding SARS on *chromosome 19 (19q13.2)*. This study investigated two Palestinian girls from the same village who presented with progressive renal failure during infancy, with atypical clinical manifestations of HUPRA syndrome including leukopenia, anemia, salt wasting, renal failure, marked hyperuricemia, hypercholesterolemia, hyperlactatemia, and hypertriglyceridemia but without pulmonary hypertension or alkalosis. Instead, they showed acidosis on routine follow-up, distinguishing them from previous cases. Using single whole exome sequencing, we identified two homozygous pathogenic variants in the SARS2 gene (c.1175A>G (p.D392G)) and (c.1169A>G (p.D390G)). These cases with their unique phenotypes, expand the SARS2 pathogenic variant spectrum and describe clinical differences between homozygous and compound heterozygous variants.

## INTRODUCTION

Hyperuricemia, pulmonary hypertension, renal failure in infancy, and alkalosis (HUPRA syndrome) is a rare autosomal recessive mitochondrial disease, first detected in 2010 in two Palestinian infants in the Jerusalem area [1]. Seryl-tRNA synthetase (SARS2) gene is a nuclear gene encoding seryl-tRNA synthetase. SARS2 gene variations will cause abnormal energy production, but given the residual activity of the mutant SARS2 gene, it can be maintained in most tissues above the necessary threshold, However, not in high-energy demanding tissues [1–3]. Therefore, may account for tubulopathy, which needs high energy to support the Na-K-ATPase cotransporter function [4]. The typical presentation for HUPRA syndrome is characterized by early-onset progressive renal failure, hyperuricemia, hyponatremia, hypomagnesemia, metabolic alkalosis, associated with systemic manifestations which include pulmonary hypertension, failure to thrive, global developmental delay, hypotonia, and ventricular hypertrophy. Additional features may include prematurity, diabetes mellitus, elevated serum lactate, and pancytopenia. In this study, two infants (Patient (I) and Patient (II)) were included in the mapping of the variated gene which is responsible for the disease.

## CASE PRESENTATIONS

### Patient (I)

The first case was the second child of healthy non-consanguineous parents of Palestinian origin. She was born at 32 weeks gestation, weighing 1.800 kg via normal vaginal delivery, and required admission to the Neonatal unit for prematurity and respiratory distress. At 5 months of age, she was admitted to the pediatric ward due to recurrent vomiting, poor weight gain, and chronic constipation of 2 months duration. Physical examination showed a dehydrated, pale, cachectic infant, with poor eye contact but normal power and tone.

Laboratory tests showed leukopenia, anemia, salt wasting, renal failure with elevated blood lactate, marked hyperuricemia, hypercholesterolemia, and hypertriglyceridemia. Abdominal ultrasound showed normal kidneys, heart echocardiography revealed no pulmonary hypertension. Moreover, Her clinical condition gradually deteriorated and within a few weeks of admission, she was in multiorgan failure, reached end-stage renal failure and regular peritoneal dialysis was initiated. At 12 months of age, the patient died after developing respiratory failure. Whole exome sequencing showed a homozygous variant in the SARS2 gene (c.1175A>G (p. Asp392Gly)), defined as a pathogenic variant according to the American College of Medical Genetics and Genomics (ACMG) classification. Laboratory data are outlined in Table S1.

### Patient (II)

The second case was the third child of healthy consanguineous parents of Palestinian descent. She was born at 36 weeks gestation, weighing 2.400 kg via cesarean section. She was admitted three times to the local hospital with vomiting, hyponatremia, and metabolic acidosis managed with fluid salt and bicarbonate. At 7 months of age, she was admitted to our pediatric ward due to poor oral intake, poor weight gain, and recurrent vomiting for the previous 3 months. Physical examination showed a dehydrated, cachectic, pale infant with good eye contact, with obvious axial and peripheral hypotonia. Laboratory tests were significant for leukopenia, anemia, hyponatremia, renal failure with elevated blood lactate, marked hyperuricemia, and hypertriglyceridemia. Abdominal ultrasound showed normal kidneys. Echocardiography revealed no pulmonary hypertension. During her admission, she developed generalized tonic clonic seizures, but had a normal CT brain. CSF analysis including herpes PCR was normal. Her symptoms were managed well with phenytoin and phenobarbital in the following months. Patient (II) required repeated blood transfusions despite receiving erythropoietin treatment. Unfortunately, she also developed progressive renal failure requiring peritoneal dialysis. Whole exome sequencing showed a homozygous variant in the SARS2 gene (c.1169A>G (p. Asp390Gly)), defined as a pathogenic variant according to the ACMG classification. Belostotsky et al. originally identified the same variant as disease-causing HUPRA syndrome, though their patient was not a relation to our Patient (II) [1]. Laboratory data are outlined in Table S1.

## MATERIALS AND METHODS

Peripheral blood samples of the proband were collected, and the genomic DNA was extracted according to the standard procedures using QIAamp DNA Bloodmini kits (Qiagen). The Illumina NOVASEQ 6000 Sequencer was used. According to the ACMG guidelines, we performed pathogenicity analysis to identify the pathogenicity of variants. “SARS2,” “HUPRA,” “mitochondrial disease,” and “seryltRNA synthase” were used as keywords to search in PubMed.

## DISCUSSION

Mitochondrial cytopathies should be considered in the differential diagnosis for unexplained renal disease in infancy [5–7]. Pathogenic homozygous variants of the SARS2 gene were detected in our two patients (c.1175A>G (p. Asp392Gly)) and (c.1169A>G (p. Asp390Gly)), in exon 13. The previously reported cases, included five homozygous variants, three of which were Palestinian (c.1169A>G), and two of which were from a Spanish family (c.1205G>A). All of the cases with the homozygous variation had typical clinical manifestations of HUPRA syndrome, two of them died of multiorgan failure and three of them died of pulmonary hypertension with a mean survival age of 17 months. Two reported Chinese cases with a new compound heterozygous variant (c.667G>A/c.1205G>A) (p.R402H) had no pulmonary hypertension or alkalosis with a survival time of 70 months. It would be important to do targeted screening and genetic counseling of families affected or at high risk of HUPRA syndrome. However, Genetic testing represents a social, political, and financial burden in our area. Unfortunately, segregation analysis was not performed on the patient’s parents to confirm the diagnosis which has crucial to decrease the incidence of genetic disorders, especially in the East Jerusalem area which is considered high-risk for high consanguinity and multiply affected individuals.

## CONCLUSION

We present the findings of two homozygous variants in the SARS2 gene, causing HUPRA syndrome with unique phenotypic characteristics. We described differing clinical manifestations arising from homozygous and compound heterozygous variants and presented a literature review of previously reported cases worldwide. Finally, renal failure is frequently observed in mitochondrial diseases, so we consider that the SARS2 gene must be one of the first-line investigations in case of unexplained renal disease during infancy.

## Supplementary Material

omcr_table_s1_laboratory_and_clinical_manifestations_in_patient_(i)_and_patient_(ii)_omad119

## Data Availability

The data used to support the findings of this study are included in the article.
