# Supplementary material for: Variants in the SARS2 gene cause HUPRA syndrome with atypical features: two case reports and review of the literature
Source: Oxf Med Case Reports. 2023 Nov 28;2023(11):omad119. doi: 10.1093/omcr/omad119 (PMC10805608; doi:10.1093/omcr/omad119)
Supplement: omcr_table_s1_laboratory_and_clinical_manifestations_in_patient_(i)_and_patient_(ii)_omad119 [file omcr_table_s1_laboratory_and_clinical_manifestations_in_patient_(i)_and_patient_(ii)_omad119.docx]

|  | **Patient ( I )** | **Patient ( II )** | **Normal Range in Infants** |
| --- | --- | --- | --- |
| Gender | female | female |  |
| ethnicity | Palestinian | Palestinian |  |
| **Renal Disease** |  |  |  |
| hyperuricemia | 16.7mg/dl | 10mg/dl | 2.5-5.5 mg/dl |
| Serum creatinine | 1.84 mg/dl | 1.37mg/dl | 0.2 - 0.4 mg/dl |
| BUN | 114 mg/dl | 52mg/dl | 5-18 mg/dl |
| Hyponatremia | 124meq/l | 128meq/l | 135 to 145 meq/l |
| hypochloremia | 85 meq/l | 96 meq/l | 96-106 meq/l |
| Progressive renal failure | + | + | - |
| Renal ultrasound | normal | normal | normal |
| **Extrarenal Manifestations** |  |  |  |
| Prematurity | + | + | - |
| Failure to thrive | + | + | - |
| Global developmental delay | + | + | - |
| Hypotonia | - | + | - |
| High lactate | 30 mg/dl | 48mg/dl | 4.5 to 19.8 mg/dl |
| Anemia | 7 g/dl | 7g/dl | 9.5 to 14 g/dl |
| Leucopenia | 2100/mm3 | 3200/mm3 | 5,000 to 19,000/mm3 |
| Platelets | 171/ µl | 197/ µl | 150-450 / µl |
| hypercholesteremia | 274 mg/dl | 290 mg/dl | 52 – 116 mg/dl |
| Hypertriglyceridemia | 1192 mg/dl | 817mg/dl | 30–105 mg/dl |
| Blood gas | PH 7.38  HCO3 20  PCO2 21 | PH 7.35  HCO3 17  PCO2 22 | PH 7.35 – 7.45  HCO3 22-26 mEq/L  PCO2 35-45 mmHg |
| Pulmonary hypertension | - | - | - |
| Ferritin | 374 ng/ml | 240 ng/ml | 7-140 ng/ml |
| SARS2 variant | c.1175A>G (p.D392G) | c.1169A>G (D390G) |  |
| Unique Phenotype | Good power and tone | Generalized tonic clonic seizures |  |

Table S1.laboratory and clinical manifestations in Patient (I) and Patient (II)
